# Serum Immunoglobulin E and Serotonin levels in Chronic Supporative Otitis Media Patients with and without treatment

**DOI:** 10.12669/pjms.37.5.2414

**Published:** 2021

**Authors:** Shafaque Mehboob, SM Tariq Rafi, Mehjabeen, Hurtimania Khan

**Affiliations:** 1 Shafaque Mehboob, Assistant Professor, Institute of Pharmacy, Jinnah Sindh Medical University, Karachi, Pakistan; 2 SM Tariq Rafi, Vice Chancellor, Jinnah Sindh Medical University, Karachi, Pakistan; 3 Mehjabeen, Dean of Pharmacy, Federal Urdu University of Arts, Science and Technology, Karachi, Pakistan; 4 Hurtimania Khan, Ear, Nose and Throat Department, Jinnah Post Graduate Center, Karachi, Pakistan

**Keywords:** Chronic Suppurative Otitis Media (CSOM), Depression, Immuonoglobulin E, Serotonin

## Abstract

**Objective:**

To determine the serum immunoglobulin E and serotonin levels of patients with chronic suppurative otitis media (CSOM) with and without treatment.

**Methods:**

This is a case-control study carried out in ENT ward of Jinnah Postgraduate Medical Centre Karachi Pakistan from May to September 2018.. Sample (n=160) was divided into four groups (40 individuals per group) as G1: control negative (group-1 without any disease), G2: positive control (patients didn’t received medicines), G3: group (patients treated with co-amoxicillin 1000mg per day) and G4: group (patients treated with ciprofloxacin1000mg per day). After treatment period of One week serum immunoglobulin E and serotonin concentrations were evaluated by Elisa method at 450nm.Statistical evaluation was carried out using one-way ANOVA (*p*<0.05) followed by post hoc (tukey test) for further group comparison. In order to find out correlation between IgE and serotonin with CSOM Pearson’s correlation was applied.

**Results:**

There was no significant (*p* > 0.05) association found between genders with serotonin as well as with IgE levels in CSOM patients. One way ANOVA showed significant difference (*p*<0.05) for IgE and serotonin levels and post hoc (tukey test) showed significant higher of IgE levels in CSOM patients of G2 positive control (diseased patients) was observed when compared to the control negative group (healthy individuals) and also from treated groups of G3 and G4. This also showed that serotonin levels were significantly low in G2 (positive control) as well as in treated groups of G3 and G4 in contrast with healthy individuals of group G1.

**Conclusion:**

Antibiotics may revert the higher levels of IgE but cannot attenuate the decreased levels of neurotransmitter (serotonin) like healthy individuals, therefore, depression levels of CSOM patients should be monitored, scored and attenuated with proper intervention of antidepressants or counseling.

## INTRODUCTION

Chronic Suppurative Otitis Media (CSOM) is a chronic middle ear inflammation commonly associated with ear discharge due to several factors such as overproduction of mucin, decrease pro-inflammatory responses and undiagnosed or untreated acute otitis media.^1^ Reported literature showed that patients with CSOM has to compromise the quality of life with other consequences of the disease such as tinnitus, vertigo, dizziness and even fever.^2^ The association between hearing loss in CSOM patients with depression, anxiety and stress was also observed in local population of Karachi.^3^

Depression may occur due to decreased serotonin levels a neurotransmitter which regulates mood elevation generated by tryptophan. It is reported that several inflammation triggering agents which are also involved in infection like bacteria, protozoa or virus can stimulate the catabolism of tryptophan (5-HTP) of the chemicals which contribute in different infections^3^ including chronic condition of otitis media is Immunoglobulin E (IgE).^4^-^6^ However, there is no study found to prove association of serotonin (decreased levels) with CSOM. In addition to this, no local study and very few researches were found regarding association of Immunoglobulin E (IgE) in CSOM patient in literature.

The object of the present study was to find association of IgE with CSOM in clinical study and to determine the serum immunoglobulin E and serotonin levels in CSOM patients with and without treatment of antibiotics which are commonly employed in to treat CSOM.

## METHODS

This is a case-control study carried out in ENT ward of Jinnah Postgraduate Medical Centre Karachi from May to September 2018. It was approved by the JPMC Ethics Committee. (Ref. No. JSMU/IRB/2017-06, dated October 23, 2017) for clinical and pre-clinical research and conducted in ear, nose and throat department of a tertiary health care hospital in accordance with the relevant regulations.

### Inclusion criteria

Patients aged between 16-60 years, belong to both the genders with or without surgical intervention having unilateral ear presentation, diagnosed with CSOM after otoscopy, medical history and physical examination with and without cholesteatoma.

### Exclusion criteria

Paediatric population and patients above 60 years with the history of neurological disorder or profound psychological distress, cardiac arrest, family history of sensorineural hearing loss (SNHL) or using hearing aid.

### Sample size calculation

the minimum sample size was calculated (n=75) with the help of OPEN-EPI, keeping 5.2% prevalence and 95% confidence interval, taking 5% margin of error but we enrolled 120 patients and 40 volunteers in the current study.^3^

Sample was divided into four groups (40 per group) as G1: control negative (group-1 without any disease), G2: group (patients didn’t receive medicines), G3: group (patients treated with co-amoxicillin ciprofloxacin1000mg per day) and G4: group (patients treated with co-amoxicillin 1000mg per day).

### Sample Collection

Blood sampling from all groups was carried out by expert technician. Serum was obtained from the blood (5ml) of the participants after centrifugation and stored -20°C in eppendorff until quantified with the help of ELIZA when sample size fulfilled.

### Determination of Immunoglobulin E (IgE) Elisa

The serum was subjected for the quantitative measurement of IgE by CalbiotechIgE ELISA Kit (catalog no. T1244), based on a solid phase sandwich assay method, based on streptavidin-biotin principle. The reading of absorbance was taken at 450 nm within 15 minutes after adding the stopping solution.

### Determination of Serotonin (St) Elisa Ki

The serum was subjected for the quantitative measurement of serotonin by Glory Science Co., Ltd ELISA Kit (catalog no. 97117) with purified human serotonin to coat microtiter plates, then add serotonin to well to proceed antibody-antigen-enzyme complex formation and measured at 450 nm.

In order to find out correlation between IgE and serotonin with CSOM Pearson’s correlation (Point-Biserial) was applied. Group comparison was done by one-way ANOVA (*p*<0.05) followed by post hoc (tukey’s test) for further group comparison. Values of IgE and serotonin were presented as mean with ±SD.

## RESULTS

Mean age of male and female patients was already reported in previous study of the enrolled patients.^3^ There was insignificant (*p* > 0.05) correlation found for gender as well as for age with serotonin and IgE levels when Pearson’s correlation (Point-Biserial correlation) test was applied in serum of CSOM patients classified into three groups in contrast with the control group of healthy people as described earlier as shown in Table-I.

**Table-I:** Pearsman’s correlation (Point-Biserial) correlation for age and gender with IgE and serotonin serum level in patients of CSOM.

Pearson Correlation forage (Point-Biserial)	Pearson Correlation	*P* value
IgE	-0.09	>0.05
Serotonin	-0.038	>0.05

Pearson Correlation for Gender (Point-Biserial)	Pearson Correlation	P value

IgE	-0.08	>0.05
Serotonin	-0.027	>0.05

*Negative and insignificant (p> 0.05).

The significant difference (p<0.05) with F-value of 48.132 was observed when one-way ANOVA was applied to evaluate the effects of CSOM on IgE with and without use of antibiotics levels. Therefore, further group comparison was done by Post Hoc (Tukey) test.

Significant higher IgE levels in CSOM patients G2 (positive control) was observed as compare to the healthy individuals G1 (negative control). However, IgE serum concentration of G1 (healthy individuals) did not show significant difference (*p*>0.05) in IgE levels as compared with G3 (ciprofloxacin treated) and G4(co-amoxicillin treated) patients. Moreover, IgE levels of G3 (ciprofloxacin treated) and G4 (co-amoxicillin treated) were significantly decreased in contrast withG2 (untreated patients) as shown in Table-II.

**Table-II:** IgE levels of G1 (control negative) from G2 (control positive), G3 (ciprofloxacin treated) and G4 (Co-amoxicillin treated) presented as mean±SD.

S. No.	Groups	IgE in male patients (IU/ml) Mean ±S.D	IgE in female patients (IU/ml) Mean ±S.D	P-value
1	G1 (Control negative)	70.13±13.1	72.43±10.22	-
2	G2 (control positive)	95.57±13.21	93.99±7.23	0.00*
3	G3 (ciprofloxacin)	75.17±5.53	71.43±9.83	1.00
4	G4 (co-amoxicillin)	81.57±6.19	87.19±10.90	0.231

* Significant difference of (p<0.05).

**Fig.1:**
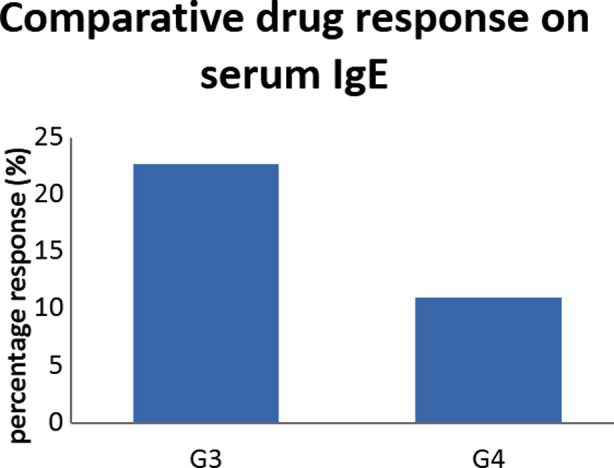
Drug response of G3 (co-amoxicillin) and G4 (ciprofloxacin) as percentage on IgE

Drug responses of both selected antibiotics were calculated taking G2 as control in order to find effects of drugs on IgE and serotonin. Fig.1 shows that G3(ciprofloxacin treated group) showed better response than G4 (co-amoxicillin treated) in reducing IgE levels in CSOM patients.

One-way ANOVA showed for serotonin levels among different groups with p=0.00 and F = 14.48. Multiple group comparison (Tukey test) showed that significant low serotonin levels in CSOM patients G2 (positive control) was observed as compare to the healthy individuals G1 (negative control). In addition to this, serotonin serum concentration of G1 (healthy individuals) also showed significant increased serotonin from G3 (ciprofloxacin treated) and G4 (co-amoxicillin treated) patients. Moreover, mean serotonin of G3 (ciprofloxacin treated) and G4 (co-amoxicillin treated) was insignificantly different as shown in Table-III.

**Table-III:** Serum serotonin levels of G1 (control negative) from G2 (control positive), G3 (ciprofloxacin) and G4 (Co-amoxicillin) presented as mean ±SD.

S. No.	Groups	Serotonin in male patients (ng/ml) Mean ±S.D	Serotonin female patients (ng/ml) Mean ±S.D	P-value
1	G1 (Control negative)	172.53±16.11	164.95±12.93	-
2	G2 (control positive)	51.38±	46.52±7.9	0.00*
3	G3 (ciprofloxacin)	117.12±14.81	96.62±12.67	0.00*
4	G4 (co-amoxicillin)	101.52±11.02	98.46±13..79	0.01*

* Significant difference of (p<0.05).

**Fig.2:**
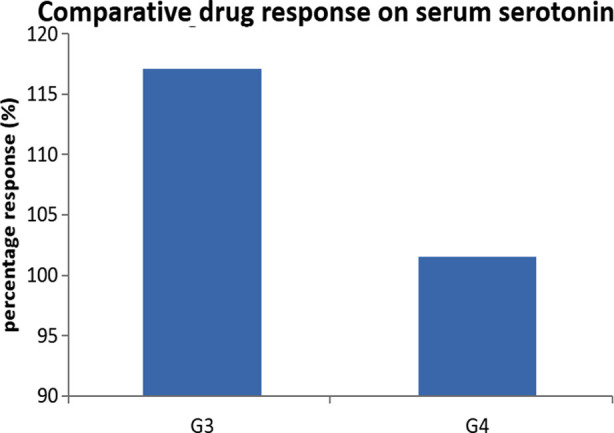
Drug response of G3 (ciprofloxacin) and G4 (co-amoxicillin) as percentage on serotonin.

Drug responses of both selected antibiotics were calculated taking G2 as control in order to find effects of drugs on serotonin. Fig.2 shows that G3(ciprofloxacin treated group) showed better response than G4 (co-amoxicillin treated) in regaining high serotonin levels in CSOM patients.

## DISCUSSION

Several contributing factors have been reported to worsen the condition of patients suffering from CSOM and resulted in different clinical presentations. Complex immune response^7^ and depression in local population of Karachi in CSOM patients,^3^ demanding researchers to pay more attention on immunoneurological studies at pre-clinical and clinical levels in this chronic ear infection.

The demographic values of the enrolled patients in current study was already reported which showed that mean age of the patients were 39.80±11.20 (G3), 43.25±9.8 (G4) and 45.3±8.7 (G2) with 1:1 ratio of male to female. Present study is the continuation of that one which showed insignificant correlation(p>0.05) found between gender as well as age with concentrations of IgE and also serotonin. This shows that both genders with CSOM are at risk to have high IgE and low serotonin levels irrespective to any particular age. However, Jarvis et al found association of age and gender with total and specific IgE in a study.^8^ Present study showed higher levels of IgE in patients suffering from CSOM (G2) as compared to the healthy group (G1). *Lasisi et al showed* role of IgE in development of chronic supportive otitis media. The study based on skin test and enzyme-linked immunoassay of the sample analysis conducted population shows that higher level of IgE in the patients’ samples indicates its role in CSOM.^8^ Therefore, present study may be helpful where no data or very few in CSOM adult patients is available in local population because IgE level may be one of the risk factors in CSOM.^9^ It was also observed that significant difference was present in IgE levels between untreated CSOM patients (G2) and treated patients with ciprofloxacin (G3) and co-amoxicillin (G4). Furthermore, there was no significant difference of IgE level between healthy group of negative control (G1) and test groups of treated patients with antibiotics (G3 and G4). This showed recovery of high IgE levels towards normalization after the antibiotics. This showed additional anti-inflammatory effects of ciprofloxacin and co-amoxicillin in decreasing IgE levels in CSOM patients as IgE is a marker of inflammation.^10^

Sachse et al.^11^ also showed anti-inflammatory effects of ciprofloxacin in decreasing interleukin 8 and Gogos et al.^12^ found that ciprofloxacin can decrease interleukin 6 concentration. Similarly, anti-inflammatory effect of amoxicillin was also established by Derartini et al.^13^ Drug response showed that ciprofloxacin (G3) and co-amoxicillin (G4) produced better response in decreasing IgE levels than co-amoxicillin. Therefore, ciprofloxacin proved better than co-amoxillin in its additional anti-inflammatory effects.

As far as serotonin is concerned, it is very important neurotransmitter which regulates mood and behavior and its low concentration is usually associated with depression and stress.^14^ In current study, group comparison showed significant decreased serotonin levels in diseased group (G2) as compared to the negative group of healthy volunteers (G1). Interesting results of serotonin were obtained which showed significant lower level of serotonin in groups maintained on antibiotics (G3 and G4) when compared with the healthy individuals (G1) but treated patients with ciprofloxacin (G3) and co-amoxicillin (G4) showed increased serotonin serum concentration than untreated CSOM patients (G2). These results showed that CSOM patients (be it treated or untreated) have serotonin depletion.

When patients got treatment with antibiotics (ciprofloxacin or co-amoxicillin) according to their pus sensitivity culture test, they showed improved and higher serotonin levels as compare to untreated group but not up to the normal level of negative control. However, both treated and untreated groups showed significant decreased serotonin levels than healthy and uninfected groups. Our previous study also showed that treated and untreated CSOM patients showed depression, anxiety and stress with high score.^3^ Therefore, serotonin may have role in behavior deficit or even pathogenesis of CSOM via tryptophan metabolism disturbance like in hepatic encephalophy.^15^ Hence, current study also showed involvement of serotonin in CSOM patient. Reported literature also supported that serotonin^16^ and other neurotransmitters disturbance may provoke other disease.^17^

Furthermore, there was no significant difference of serotonin level between treated patients of ciprofloxacin (G3) as compare to co-amoxicillin (G4). However, drug response showed that ciprofloxacin (G3) and co-amoxicillin (G4) produced better response in increasing serotonin levels than co-amoxicillin.

Overall, antibiotics helped to regain normal IgE levels but not serotonin, leaving patients in depression and stress. This issue needs to be solved by health caretakers either by counseling with patients or with antidepressant intervention. Hence, CSOM patients need to be scored for depression and properly treat so that they get rid of not only from infection but also from depression.

### Limitation of the study

The study was limited to certain number of patients and volunteers but it should be conducted at larger scale with intervention of patients’ counseling or antidepressant for patients who are at higher risk of serotonin depletion.

## CONCLUSIONS

CSOM induced immunoglobulin E levels can be controlled with antibiotic treatment of this chronic infection but serotonin cannot be normalized even after ciprofloxacin or co-amoxicillin therapy. Therefore, patients with CSOM should be properly monitored for depression, anxiety and stress and subjected to psychological therapy if require.

### Authors’ Contribution:

**SM** Designed, collection of data, analysis & interpretation of data. He is also responsible and accountable for this study.

**SMTR** Supervise and final approval of the version.

**M** Did edit of manuscript.

**HK** Did Data collection.
